# Antitumor effects of a lectin purified from *Genipa americana* in human cancer cell lines and murine melanoma cells

**DOI:** 10.3389/fonc.2026.1913802

**Published:** 2026-08-05

**Authors:** Ricardo Bezerra Costa, Monizy da Costa Silva, Emisael Stênio Batista Gomes, Rogério Gonçalves da Rocha, Stella Freitas de Queiroz Fragoso, Marta Angelo dos Santos, Ana Kelly da Silva Fernandes Duarte, André Luiz Sena Guimarães, Carlos Alberto de Carvalho Fraga, Francis Soares Gomes

**Affiliations:** 1 Instituto de Química e Biotecnologia, Federal University of Alagoas, Maceió, Alagoas, Brazil; 2 Health Science Laboratory, State University of Montes Claros, Montes Claros, Brazil; 3 Laboratory of Histopathology, Federal University of Alagoas (UFAL), Arapiraca, Alagoas, Brazil

**Keywords:** carcinoma, caspase-3, E-cadherin, lectin, melanoma

## Abstract

**Background:**

Plant lectins have attracted considerable interest because of their selective interactions with tumor-associated glycans and their potential antitumor properties. However, the effects of the lectin isolated from the bark of *Genipa americana* (GaBL) on different cancer cell models remain unknown. Therefore, this study aimed to evaluate the effects of GaBL on the phenotypes of human epidermoid carcinoma (A431), human tongue squamous cell carcinoma (SCC9), and murine metastatic melanoma (B16) cells.

**Methods:**

Cancer cell lines were treated with 10 µg/ml of GaBL to assess cell viability, cell migration and invasion, as well as the identification of cell membrane alterations associated with apoptosis. Real-time polymerase chain reaction for caspase-3 was performed to verify if apoptosis is activated by lectin treatment. The mRNA expression of proteins (E-cadherin, type I collagen) related to the epithelial-mesenchymal transition was also analyzed.

**Results:**

GaBL decreased (27.5-50%) cell proliferation and reduced cell migration in all strains evaluated. Additionally, the lectin decreased the invasion of SCC9 cells. Apoptosis was higher against B16 and SCC9 cells treated with the lectin. GaBL induced increased mRNA expression of caspase-3, E-cadherin, together with reduced type I collagen mRNA expression in the evaluated cell lines. GaBL inhibits growth, migration, and invasion of human head and neck cancer cells and modulates the expression of genes associated with apoptosis and epithelial-mesenchymal transition.

**Conclusion:**

Our findings identify GaBL as a promising bioactive molecule with antitumor activity *in vitro* and support further studies to elucidate its molecular mechanisms and evaluate its therapeutic potential *in vivo*.

## 1 Introduction

In 2026, the American Cancer Society reported a significant number of cases and deaths from cancer. According to the publication, in 2026 the United States incidence of cancer will be approximately 2.11 million new cases and 626,140 deaths, and these numbers tend to increase 16% to 35% for metastatic melanoma (1). Cancer metastasis is the primary cause of cancer mortality, accounting for about 90% of cancer deaths. Metastasis is dependent on several steps, including vessel formation, cell adhesion, migration, invasion, and cell proliferation (2). It occurs when genetically unstable neoplastic cells adapt to a tissue microenvironment far from the primary neoplastic mass. This process involves the selection of pathways advantageous for neoplastic cells and the ability to recruit stromal pathways that accommodate these cells (3).

Due to this heterogeneity, cancer treatment is still fraught with limitations. Cytotoxic chemotherapy, one of the main forms of treatment, is limited by cell resistance to drugs, in addition to causing many adverse side effects. Thus, tumors are highly adaptable, as they can activate resistance signaling routes and inactivate the death signaling cascade, leading to resistance to treatment (4–6). There is a need to develop new therapeutic strategies that are more selective in targeting specific pathways, sparing healthy cells from exposure to these drugs.

Plant-derived compounds have been extensively investigated for cancer prevention and for the development of novel anticancer therapies, as many natural products exhibit lower toxicity than conventional chemotherapeutic agents (7). Among the studied compounds in a plant extract, there are lectins, proteins that have high specificity in carbohydrate recognition and can bind in a reversible way to monosaccharides or oligosaccharides through the non-catalytic domain (8, 9). Due to the wide structural diversity, these biomolecules have several biological activities, such as anti-inflammatory, antibacterial, antiviral, antifungal, immunomodulatory, and antitumor properties (7, 8, 10–14).

Some lectins have greater affinity for carbohydrates found in tumor cells than for those present in normal cells. Therefore, lectins can be used as effective anticancer agents and/or as biomarkers. Several studies use plant lectins to identify malignant cancer cells through the analysis of glycosylated patterns or by using microarrays of these proteins (9, 11, 15). These molecules are also capable of reducing proliferation and inducing tumor cell apoptosis, migration, and invasion and can be used in the treatment of malignant neoplasms (16–20).

Barks of genipap (*Genipa americana* L.) have a lectin (GaBL) with a molecular mass greater than 200 kDa. GaBL has broad stability at pH and temperature, remaining stable in temperature and pH values found in the human body. It was also detected that GaBL showed high specificity to lactose and fetuin glycoprotein (21). Increased level of fetuin is described as a potential marker for metastatic disease diagnosis (22–24). Considering the characteristics presented by this isolated biomolecule, along with the descriptions of the use of this plant as a phytotherapeutic product, the objective of the present work was to evaluate the effects of the lectin from the bark of *G. americana* on the human skin cancer cell line (A431), melanoma (B16), and tongue squamous cell carcinoma (SCC9) phenotypes.

## 2 Materials and methods

### 2.1 Collection of plant material and protein purification

The bark of genipap (*G. americana*) was collected in Coruripe, on the southern coast of Alagoas, Brazil (10°07′32″ S, 36°10′32″ W), with the authorization of the National System of Genetic Heritage and Management of Associated Traditional Knowledge (SISGEN) (process number A87C1CC) and one sample was sent to the Environmental Institute of Alagoas (IMA) for identification, obtaining registration number 64576. The plant material was stored, in plastic packaging, and taken to the Laboratory of Metabolism and Proteomics - LAMP, at the Federal University of Alagoas.

GaBL was isolated as described by Costa et al. (21). The bark, after being dried at room temperature, was weighed and ground. Then, 1:2 extraction buffer (50 mM Tris-HCl, pH 8.0) was added and the solution was kept under stirring for 16 hours. After this time, the solution was filtered and centrifuged at 15,000g, for 15 minutes, at 4 °C, using a refrigerated centrifuge (HERMLE-Z236K). The supernatant was collected and stored at – 20 °C.

For the purification process, the supernatant obtained during the extraction procedure was treated with ammonium sulfate at 20% saturation, for 4 hours, followed by centrifugation at 15,000 g, at 4 °C, for 15 min. The precipitate was resuspended in Tris-HCl buffer pH 8.0, 50 mM and dialyzed against distilled water (4 h) and 50 mM Tris-HCl pH 4.0 (2 h).

The precipitated fraction was subjected to liquid chromatography with a molecular exclusion column, composed of a stationary phase formed by Sephacryl S-100 HR resin (16 mm x 60 cm), equilibrated with 50 mM Tris-HCl buffer pH 8.0, with 0.5 M NaCl as a mobile phase. The flow was adjusted to 0.1 mL/min, with a total column volume of 50 mL, connected to a fast performance liquid chromatography system (AKTA purifier 25 M1, General Electric). A volume of 300 µL of the precipitate resuspended in Tris-HCl buffer pH 8.0, 50 mM was applied and 2 mL per fraction were collected, which were evaluated for protein concentration and hemagglutinating activity (HA) and the active fractions were called GaBL.

### 2.2 Protein concentration and hemagglutinating activity assay

Protein concentration was estimated according to Bradford (25), using bovine serum albumin (0.009–250 μg/mL) as a standard. The HA assay was performed on 96-well microtiter plates using a suspension of rabbit erythrocytes (2.5% v/v) previously treated with glutaraldehyde, as previously described by Silva et al. (26). For this purpose, a serial dilution of 50 µL of the sample was performed (in duplicate) in 0.15 M NaCl in a row of the plates and, immediately afterwards, 50 μL of the erythrocyte suspension was added. The number of units of HA corresponded to the reciprocal of the highest dilution of the sample that promoted agglutination of erythrocytes. Specific HA was defined as the ratio between HA and protein concentration (mg/mL). The erythrocyte collection was approved by the Animal Experimentation Ethics Committee of the Federal University of Pernambuco (process 23076.033782/2015-70).

### 2.3 Cell culture and treatment

The immortalized cell line of tongue squamous cell carcinoma (SCC9) (ATCC, Manassas, VA, USA), the human epidermoid carcinoma cell line A431 (ATCC^®^ 1555™, USA) and murine metastatic melanoma cells of the B16 line, provided by the Federal University of Minas Gerais, were used. B16, A431 and SCC-9 cells were cultured at 37 °C in 5% CO_2_ in a Ham’s F-12 nutrient mixture (Invitrogen, Carlsbad, CA, USA) supplemented with 400 ng/ml hydrocortisone (Sigma, St. Louis, MO) and 10% fetal bovine serum. Although these cell lines originate from different tissues and species, they were selected to provide an initial evaluation of the antitumor activity of GaBL in distinct experimental models commonly used in cancer research. Mouse embryonic fibroblasts (3T3 cell line) were kept in medium Dulbecco’s modified Eagle medium, containing 10% fetal bovine serum, L-glutamine (2 mM) and gentamicin (40 μg/mL) in incubator at 37 °C and 5% CO_2_.

### 2.4 Cell viability assay

To explore the cytotoxic activity of GaBL to noncarcinogenic cells, the colorimetric MTT (3-(4,5-dimethylthiazol-2-yl)-2,5-diphe-nyltetrazolium bromide) assay was pursued with fibroblastic (3T3 cell line), with an incubation period of 24 and 48h at 37 °C in a CO_2_ humidity incubator (27). A 96-well plate at a density of 1 × 10^4^ cells/well were treated with the serial concentrations of the compound GaBL (0.5, 1, 5, 10, and 50 μg/ml). At the end of the treatment, 5 mg/mL of MTT (Sigma-Aldrich – EUA) in phosphate-buffered saline was added, and the cells were incubated for another 4 h. Dimethyl sulfoxide (200µl) was added to each well after removal of the supernatant. After shaking the plate for 10 min, cell viability was assessed by measuring the absorbance at 490 nm using an Enzyme-labeling instrument (EX-800 type). All measurements were resulted of an experiment performed in triplicate. Cell viability was expressed as a percentage of untreated cells (control, 100%). The cytotoxic effect was considered when the highest concentration gave a response that was < 70% of the control.

The cytotoxic effect against tumor cells (SCC9, A431 and B16 cell lines) was evaluated through MTT assay. A 96-well plate at a density of 1 × 10^4^ cells/well, cultured for 12 h under 37 °C in 5% CO_2_, was treated with 10 μg/ml of GaBL for 24 h. Cell growth curve was completed using time as the abscissa and A value (mean ± standard errors) as the ordinate.

### 2.5 Cell migration assays

Cell migration was monitored in a wound scratch assay as described previously (28). Briefly, a scratch was made in the center of the well with a sterile pipette tip in a confluent cell layer and washed twice in phosphate-buffered saline to remove debris and smooth the edge of the scratch. Then, fresh medium was added to the cells with or without GaBL (10 μg/ml). The plates were then incubated at 37 °C in 5% CO_2_ for up to 24 h. Wells were photographed at the beginning of the experiment and after 24 hours. Pictures were obtained with a camera SC30 (Olympus, Center Valley, PA, USA) in an IX81 inverted microscope (Olympus, Center Valley, PA, USA). ImageJ software was used for analysis (29). To calculate the wound healing ratio, the initial area (in pixels) was divided by the final cell-free area (in pixels). Each experiment was carried out in triplicate.

### 2.6 Invasion assay

For the transwell invasion assay, a polycarbonate membrane with pores of 8 μm in diameter was used for the upper chamber covered with 50 μl of Matrigel (BD Biosciences, USA) as previously described (30). Then, in the top chamber, 2×10^5^ SCC9 cells were plated onto each well and treated with 10 μg/ml of lectin. In lower compartment, 10% fetal bovine serum was added as a chemoattractant factor for cells. The multiwell plate was covered and incubated for 24 h at 37 °C in CO_2_ incubator. Then the upper chamber was inverted to remove both cells that had not invaded and culture medium from the upper chamber. Only the cells that had migrated were fixed in 4% formaldehyde in PBS and stained with 2% Crystal Violet. Images were acquired at a scale of 200 μm with the camera SC30 (Olympus) in an IX81 inverted microscope (Olympus). The number of cells that invaded was determined through counting. The experiment was performed in triplicate.

### 2.7 Cell apoptosis assay

Dual acridine orange/ethidium bromide (AO/EB) fluorescent staining, visualized under a fluorescent microscope, can be used to identify apoptosis-associated changes of cell membranes during the process of apoptosis (31). This method can also accurately distinguish cells in different stages of apoptosis. Dual AO/EB staining was used to examine apoptosis in a human oral cancer cell line treated with GaBL (10 μg/ml). The samples in a 96-well plate were divided into 2 groups, with 24 well samples in each group. Dual fluorescent staining solution (1 µl) containing 100 µg/ml AO and 100 µg/ml EB (AO/EB, Sigma, St. Louis, MO) was added to each well. The morphology of apoptotic cells was examined after 24h and 500 cells were counted within 20 min using a fluorescent microscope. Intense EB (Ex360-370nm, Em420-460nm, filter DM400) staining indicates cell death, while extreme AO (Ex460-495nm, Em510-550nm, filter DM505) indicates live cells. Dual acridine orange/ethidium bromide (AO/EB) staining method was repeated three times.

### 2.8 Analyses of gene expression

To verify the effect of lectin treatment on gene expression in A431, B16 and SCC9 cells, RNA was isolated from tumor tissue using the Trizol reagent (GIBCO Invitrogen), according to the manufacturer. Total RNA was reverse transcribed, and the subsequent cDNA was heated at 95 °C to terminate the reaction. For real-time polymerase chain reaction (PCR), 2 µl of the cDNA was added to TaqMan Universal PCR Master Mix (Applied Biosystems) with the E-cadherin, Collagen type 1 alpha 1 (Col1A1), and Caspase-3 specific primer/probe set (Applied Biosystems) as described in Supplemental digital content 1(**Supplementary Table 1**).

Amplification was performed on an ABI 7000 Sequence Detection System. All reactions were done in triplicate, and 18S rRNA served as an internal control. The results were quantified as Ct values, where Ct was defined as the threshold cycle of PCR at which the amplified product is first detected and defined as relative gene expression (the ratio of target/control).

### 2.9 Statistical analysis

All experiments were conducted in triplicate. Analyses were performed using GraphPad Prism software (Version 5.0, GraphPad Software Inc., San Diego, CA, USA). Data were presented as the mean ± standard error of the mean (SEM). Comparisons among multiple groups were performed using one-way analysis of variance (ANOVA), followed by Dunnett’s multiple comparisons test. Comparisons between two groups were performed using the unpaired two-tailed Student’s *t*-test. Differences were considered statistically significant when *P* < 0.05.

## 3 Results

### 3.1 Lectin isolation

The crude extract showed a specific HA of 640 and was subjected to ammonium sulfate fractionation. Chromatography of the dialyzed supernatant fraction (specific HA = 1561) on a Sephacryl S-100 HR column yielded purified GaBL with a specific HA of 1838, corresponding to a 2.9-fold purification. These purification parameters are consistent with those previously reported by Costa et al. (21), who isolated GaBL using the same purification strategy.

### 3.2 Effect of lectin on cell viability

**Figure 1:**
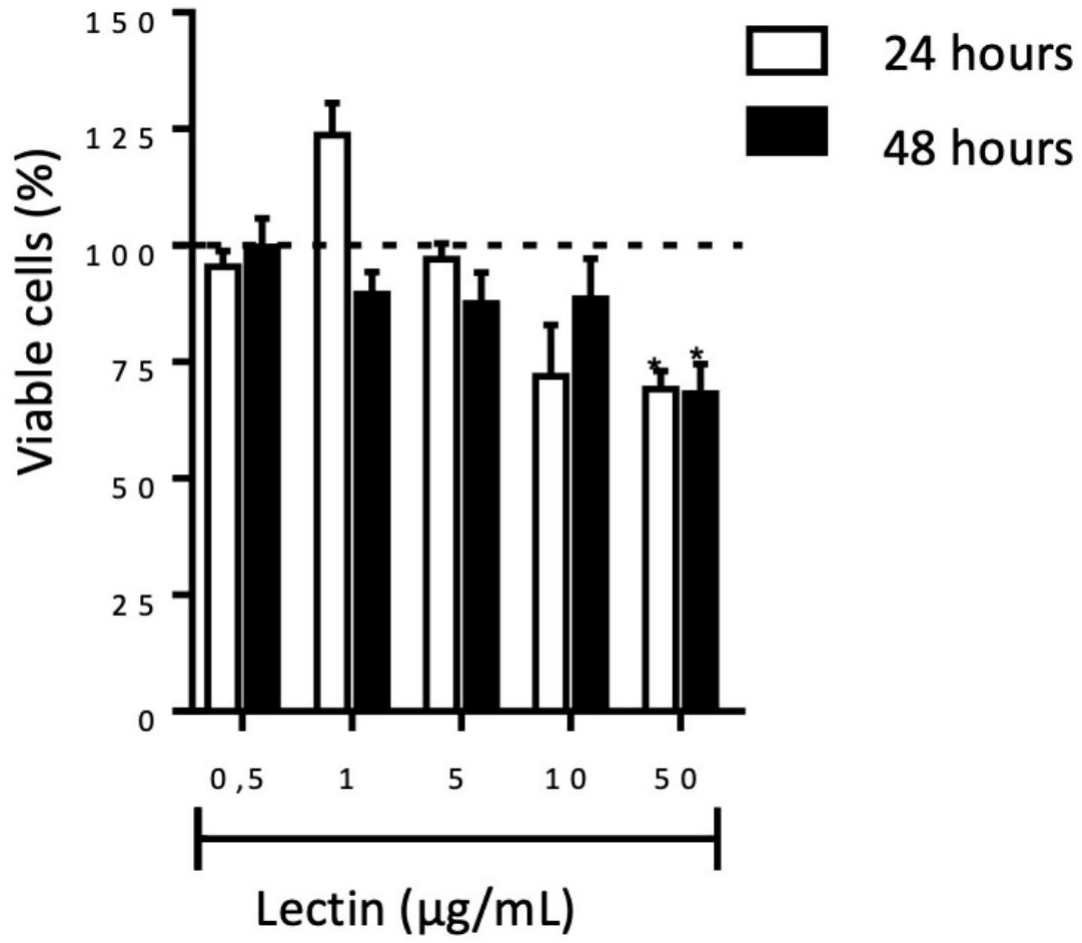
Effect of the lectin (GaBL) on the viability of fibroblast 3T3 cells assessed by the MTT assay. Cells were seeded in 96-well plates at a density of 1 × 10^4^ cells/well and treated with different concentrations of GaBL (0.5, 1, 5, 10, and 50 μg/mL) for 24 h (white bars) and 48 h (black bars). Untreated cells were used as controls, and cell viability was expressed as a percentage of the control group (100%). The cytotoxic effect was considered when cell viability was reduced below 70% of the control value. Data are presented as mean ± SD of triplicate measurements. Statistical comparisons among multiple groups were performed using one-way ANOVA followed by Dunnett’s multiple comparisons test. *p < 0.05 compared with the respective untreated control group.

GaBL was investigated for cytotoxicity on normal fibroblast cell line (3T3) before the evaluation of antitumoral activity since compounds with unselective cytotoxicity are not viable for use in chemotherapy. Treatment for 24 h with pure lectin at concentrations of 0.5, 1, 5 and 10 µg/mL did not change fibroblast viability (**Figure 1**). However, cells treated with 50 µg/mL of the lectin showed a 31% (p < 0.05) reduction in cell viability. A similar effect on viability was observed with the 48 h treatment with the lectin (p < 0.05). Therefore, the concentration of 10 µg/mL was used for the remaining tests.

**Figure 2:**
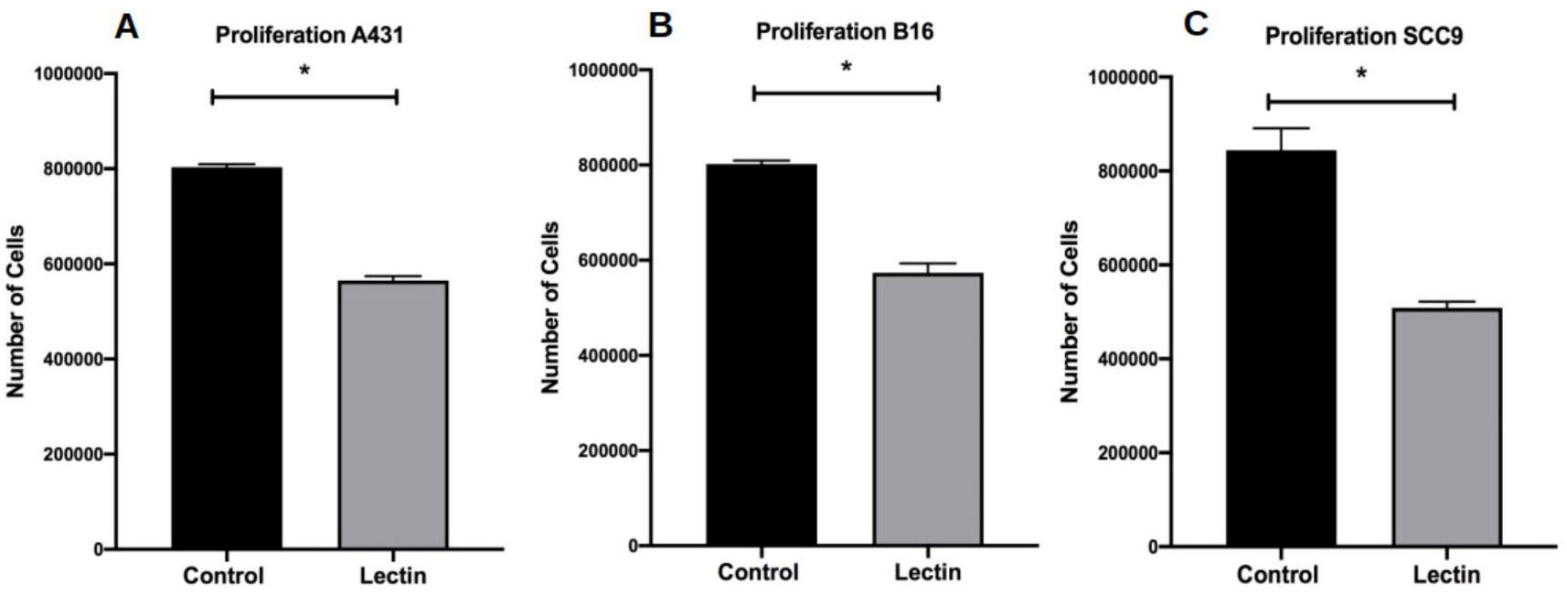
Effect of the lectin (GaBL) on the viability of A431, B16, and SCC9 cancer cells. Each panel **(A, B, C)** shows higher cell numbers in control compared to lectin-treated groups, with significant differences indicated by asterisk symbols. Cells were treated with GaBL (10 μg/mL) for 24 h. Untreated cells were used as controls, and cell viability was expressed as a percentage of the control group (100%). Data are presented as mean ± SD of triplicate measurements. Statistical comparisons between treated and control groups were performed using the unpaired two-tailed Student’s *t*-test. *p < 0.05 compared with the respective untreated control group.

To elucidate whether lectin contributes to the viability of tumor cell lines, MTT assay was performed on the A431, B16 and SCC9 cells treated with 10 μg/ml of lectin for 24h. The lectin significantly decreased the percentage of treated cells. After treatment with lectin, the viability was decreased in both A431, B16, and SCC9 (**Figure 2**). These findings indicate that GaBL decreases the viability of tumor cells, which is consistent with its antiproliferative activity.

### 3.3 Effects of lectin on migration and invasion

**Figure 3:**
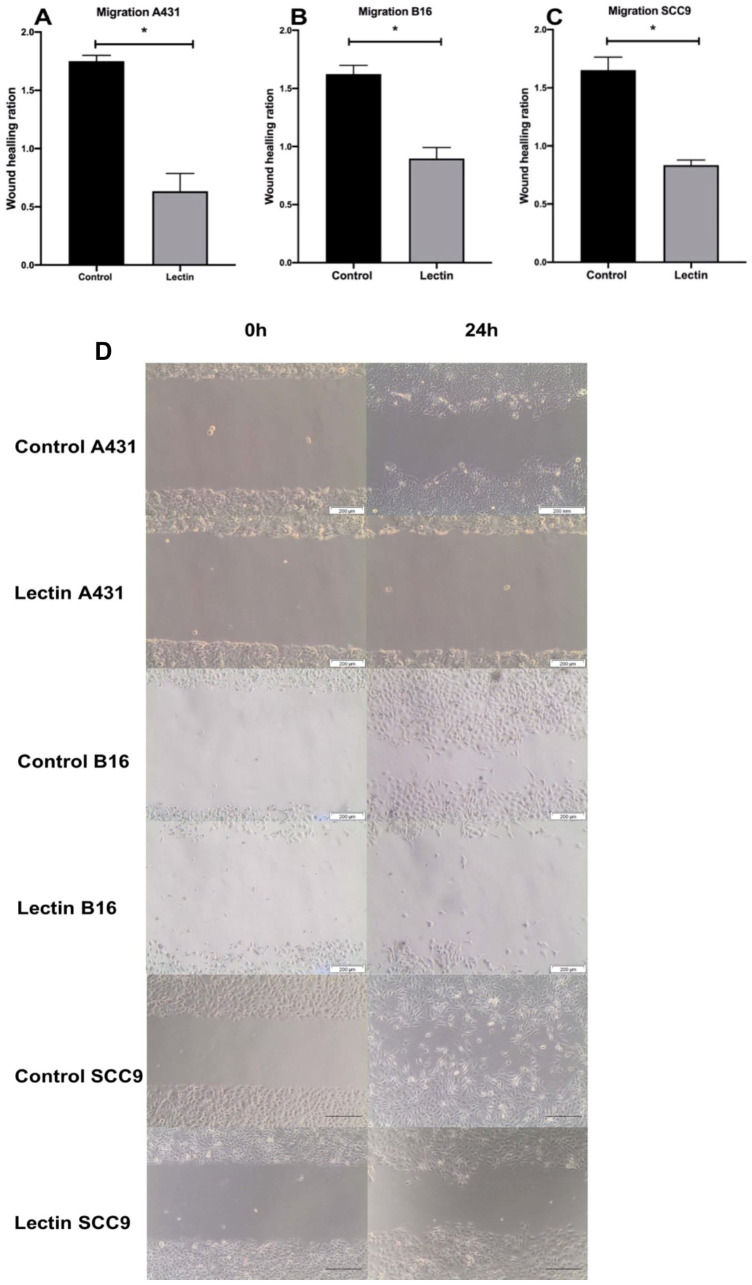
Effect of the lectin (GaBL) on the migration of A431 **(A)**, B16 **(B)**, and SCC9 **(C)** cells, assessed by the wound healing assay, showing significantly lower migration in lectin (10 μg/mL) treated groups compared to controls. Significant differences were indicated by asterisk symbols. Below, panel **D** presents microscope images of wound healing assays for each cell line and treatment at zero and twenty-four hours, demonstrating reduced cell migration in lectin-treated samples compared to controls over time. Scale bar = 200 μm.

Wound-scratch assay was performed on the cancer cells. Cell migration assays were performed with confluent monolayers of human A431, B16, and SCC9 cells treated with GaBL, which were mechanically scratched and subsequently allowed to re-populate the wounded cell-free areas. Application of lectin resulted in a significant decrease of scratch area closure, consistent with decreased cell migration against all tumor cells evaluated (**Figure 3**).

**Figure 4:**
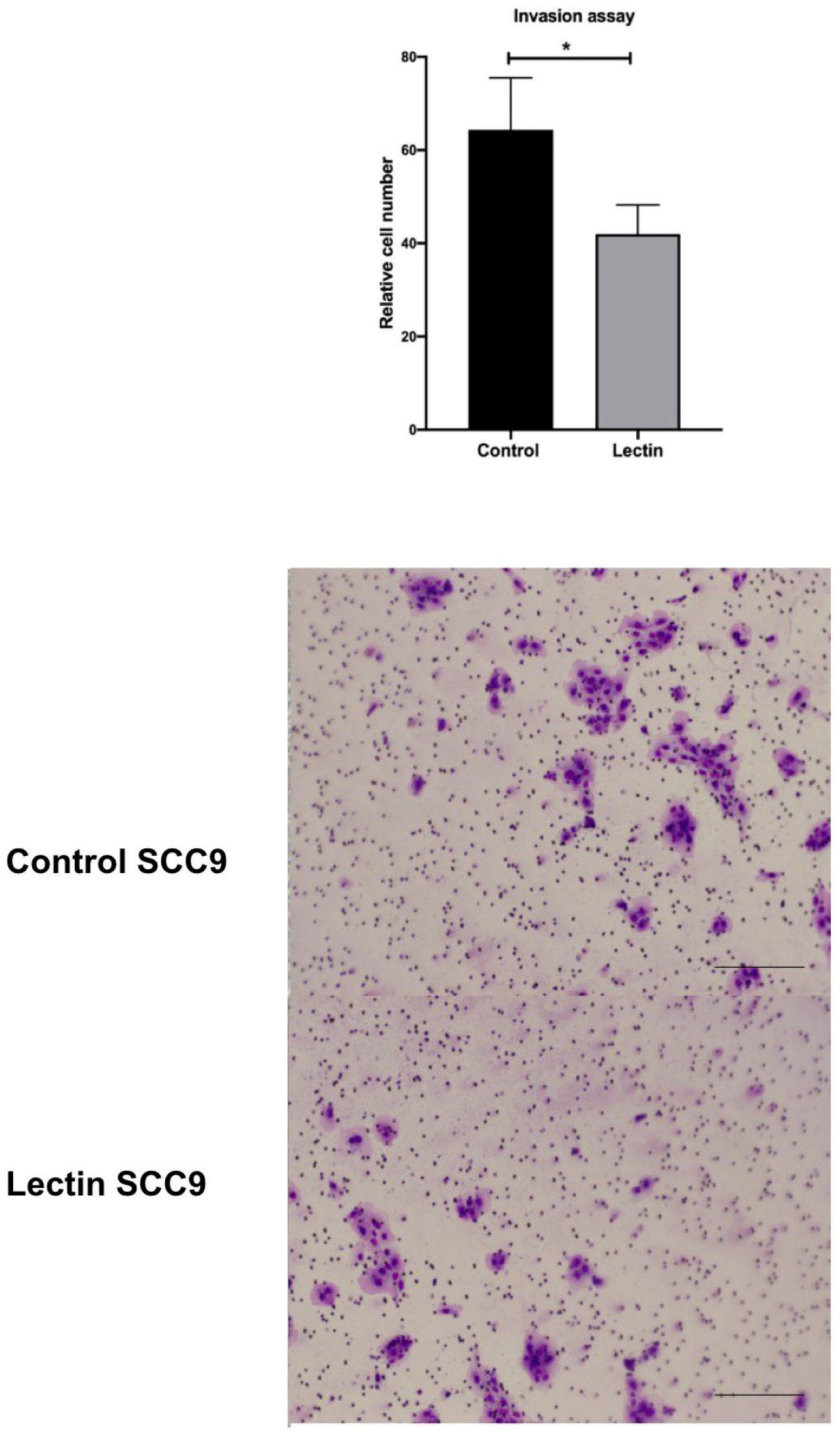
Effect of the lectin (GaBL) on the invasiveness of SCC9 cells assessed by the Matrigel-coated Transwell invasion assay. SCC9 cells were treated with GaBL (10 μg/mL) or left untreated (control) for 24 h. The bar graph shows significantly lower relative cell number in the lectin group. Significant differences were indicated by asterisk symbols. Microscopy images depict more purple-stained invasive cells in control SCC9 and visibly fewer in lectin SCC9. Scale bar = 200 μm.

Invasiveness is an important characteristic of cancer cells and a target for the development of anticancer agents. According to matrigel invasion assay, lectin significantly reduced invasiveness in SCC9 treated cancer cells (**Figure 4**).

### 3.4 Effect of lectin on apotosis and on caspase-3, E-cadherin, and Col1A1 expression

**Figure 5:**
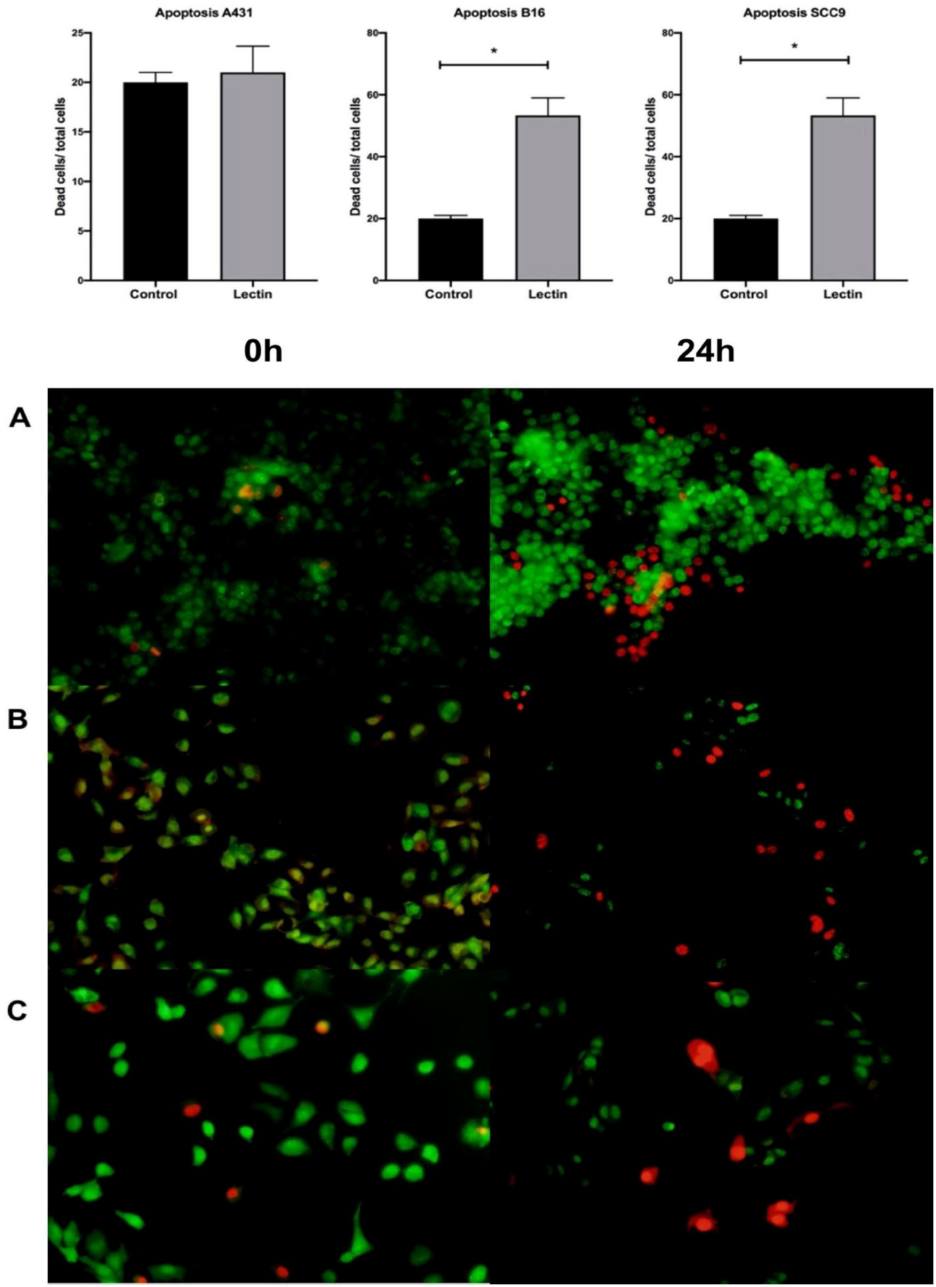
Effect of the lectin (GaBL) on cell death morphology assessed by acridine orange/ethidium bromide (AO/EB) staining. Three vertical bar charts on top display the percentage of dead cells among total cells for A431, B16, and SCC9 lines, comparing control and lectin (10 μg/mL) treatment. B16 and SCC9 show significantly increased apoptosis with lectin, indicated by an asterisk. Below, fluorescent microscopy images **(A)** (A431), **(B)** (B16), and **(C)** (SCC9) show green live cells and red dead cells at zero and twenty-four hours. Images on the right (twenty-four-hour condition) show a visibly increased number of red dead cells compared to those on the left (zero-hour condition) for B16 and SCC9 cell lines.

Acridine Orange/Ethidium Bromide Cell death assay reveals that the lectin significantly increased the number of apoptotic cells when compared to control, specifically in B16 and SCC9 cancer cells (**Figure 5**).

**Figure 6:**
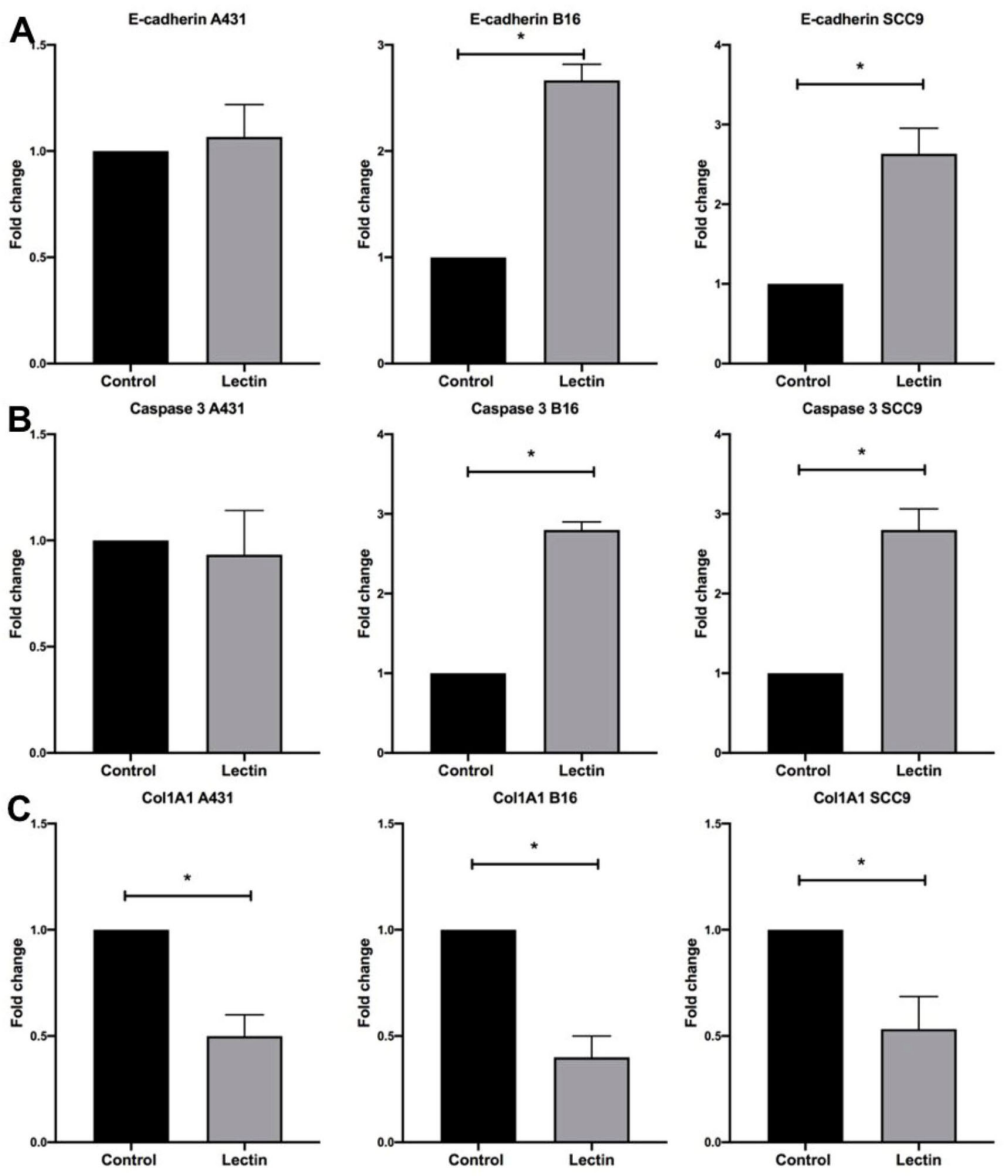
Effect of the lectin (GaBL) on the mRNA expression of E-cadherin **(A)**, caspase-3 **(B)**, and collagen type I alpha 1 (Col1A1) **(C)** in A431, B16, and SCC9 cells determined by quantitative real-time PCR (RT-qPCR). Cells were treated with GaBL (10 μg/mL) or left untreated (control). Each compares control and lectin treatment, with significant differences marked by asterisks in specific panels.

To investigate whether the observed increase in apoptotic cells was associated with transcriptional regulation of apoptosis-related genes, a reverse transcription-PCR for caspase-3 was performed. The lectin increased caspase-3 transcription when compared with non-treated B16 and SCC9 cells. In A431 cells, the lectin did not alter caspase- 3 mRNA levels. Additionally, a quantitative real-time PCR was performed to evaluate the mRNA expression levels of epithelial-to-mesenchymal transition-related proteins (E-cadherin and Col1A1). GaBL induced the upregulation of E-cadherin and the suppression of Col1A1 in both A431, B16, and SCC9 cells (**Figure 6**).

## 4 Discussion

In a previous study, it was demonstrated that *G. americana* L. has a lectin called GaBL with wide stability in pH and temperature. This lectin was not inhibited up to 120 °C, losing its activity only at pH 2. GaBL is also able to bind to various carbohydrates and glycoproteins. These characteristics make GaBL a desirable candidate for potential use in the treatment of infectious diseases in humans (21).

Viable biomolecules in cancer therapy have become a promising target in the field of biotechnology. Carbohydrate-specific binding proteins play an important therapeutic and biotechnological role (32). Lectins can recognize carbohydrate or glycoconjugate fractions and bind reversibly to cancer cells with high specificity and without changes in the covalent structure (33). The commercially acquired lectins DBA, LCA, PHA-L, and WGA showed high recognition for cancer cells through binding to glycans present on the cell surface (34). In another study, Freitas et al. (35) described that the lectins E-PHA and L-PHA bind to cancer-related N-glycans, and the AAL lectin binds to fucose glycans. It is important to highlight that glycoconjugates present in extracellular vesicles play versatile roles in cancer (32). A study by Surman et al. (36) showed that a group of lectins can bind specific glycan epitopes in extracellular vesicles compared to parental cell membranes. It is noteworthy to describe that, to the best of our knowledge, this is the first study showing the association between this lectin (GaBL) and cancer cells.

In this study, it was observed that GaBL did not show cytotoxicity for normal fibroblast cells. However, GaBL had an antiproliferative effect on cancer cells (A431, SCC9, and B16). Although these cell lines originate from different tissues and species, they were selected to provide an initial evaluation of the antitumor activity of GaBL in distinct experimental models commonly used in cancer research. Therefore, the objective of this study was not to directly compare their biological responses, but rather to determine whether GaBL exhibits antitumor activity across different tumor models. The biological effects observed in the MTT assay were accompanied by reduced migratory and invasive capacities, as well as increased apoptotic morphology, indicating that GaBL exerts multiple antitumor effects beyond alterations in cellular metabolic activity alone. These findings were supported by the mRNA expression levels of E-cadherin and Col1A. These data are consistent with the literature regarding the roles of some described plant lectins with antitumor action, such as DVL, a lectin purified from *Dioclea violacea* seeds, which showed cytotoxic activity against human cells, acting on mechanisms of cell migration and proliferation (37). The lectin from *Canavalia ensiformis* seeds (ConA) exhibited antitumor action, capable of inhibiting the proliferation of cancer cells, reducing tumor growth, and inducing apoptosis in tumor cells (16). Although only a single concentration of GaBL was evaluated in the functional assays, this concentration was selected based on its lack of cytotoxicity toward normal fibroblasts. Future studies should include dose-response analyses to determine IC_50_ values, define the therapeutic window, and further characterize the antitumor activity of GaBL.

The increase in E-cadherin expression is important in regulating cell adhesion and maintaining tissue integrity, preventing disintegration and possible metastasis *in vivo*. In this work, it was observed that E-cadherin expression was increased by lectin treatment, while Col1A1 was reduced. It has been demonstrated that the loss of E-cadherin expression/function is associated with the development and progression of different types of cancer. The loss of E-cadherin is often associated with epithelial-mesenchymal transition (EMT) and a more aggressive cancer phenotype (38). Both the positive regulation of Col1A1 and the reduction or loss of E-cadherin expression are considered characteristics of EMT, a complex developmental program that allows carcinoma cells to suppress their epithelial characteristics and shift to mesenchymal ones (39, 40). These transcriptional changes showed by GaBL (positive regulation of E-cadherin and negative regulation of Col1A1) are compatible with a less invasive phenotype and are consistent with modulation of EMT-related pathways. However, because EMT is regulated by multiple molecular pathways, additional studies evaluating a broader panel of EMT markers at both the gene and protein levels are required to confirm whether GaBL directly affects this process.

Previous studies have shown that some plant lectins can activate signaling pathways such as EGFR mediated by Ras-Raf-MEK-ERK, leading to apoptosis and autophagy in cancer cells (41). Although these observations suggest potential mechanisms through which lectins may exert antitumor activity, the involvement of these signaling pathways in the biological activity of GaBL remains to be experimentally investigated. Mistletoe lectins (MLs) have antiproliferative mechanisms, being potent cytotoxic anti-tumor lectins and inducing apoptosis by efficiently inhibiting protein synthesis in acute lymphoblastic leukemia and hepatocarcinoma cells (42, 43). In this study, it was observed that the lectin (GaBL) induced the death of cancer cells. The data indicated that treatment with the lectin (GaBL) markedly triggered caspase-3 transcription. Although increased caspase-3 mRNA expression does not necessarily reflect caspase activation at the protein level, these findings, together with the morphological evidence obtained by Acridine Orange/Ethidium Bromide staining, support the hypothesis that GaBL promotes apoptotic cell death. A study conducted by Batcha et al. (44) showed that the lectin extracted from bananas (BanLec) triggered apoptosis, manifesting through distinct morphological changes. These included the formation of bubbles in the cell membrane, reduction in cell volume, chromatin condensation, cell budding, and nucleus fragmentation. Similarly to GaBL, the expression of the caspase-3 gene was notably positively regulated, clearly suggesting the induction of the apoptotic process during treatment with BanLec.

The lectin from the tuberous rhizome of *Kaempferia rotunda* also exhibited anticancer activity against SW480 and SW48 colon cancer cells, acting on the activation of caspase-3 and caspase-9, preventing cell proliferation, and inducing the apoptotic process in the mitochondrial intrinsic pathway (45). The lectin isolated from the hemolymph of *Gluttonous beauts* exerted cytotoxic effects *in vitro* on human breast cancer cells (MCF-7). This lectin can induce apoptosis by activating caspase-3 (46). Apoptosis is an important mechanism in controlling cell proliferation as part of normal development.

## 5 Conclusion

GaBL exhibits antiproliferative and anti-migratory activities against the A431, B16, and SCC9 cancer cell lines. The lectin also showed anti-invasive activity and modulates the expression of genes associated with apoptosis and epithelial-mesenchymal transition in some of the tested cell lines. Further studies involving protein-level analyses, caspase activity assays, and the evaluation of additional EMT markers are warranted to confirm the underlying molecular mechanisms. These findings provide the first evidence of the antitumor potential of GaBL and support further dose-response and *in vivo* studies.

## Data Availability

The datasets presented in this study can be found in online repositories. The names of the repository/repositories and accession number(s) can be found in the article/**Supplementary Material**.
