# Supplementary material for: Antitumor effects of a lectin purified from Genipa americana in human cancer cell lines and murine melanoma cells
Source: Front Oncol. 2026 Aug 5;16:1913802. doi: 10.3389/fonc.2026.1913802 (PMC13485777; doi:10.3389/fonc.2026.1913802)
Supplement: Supplementary file 1 [file Table1.docx]

Table S1: Primer used in real-time PCR

| Primers for Real-time PCR |  | Sequence (5’ 3’) |
| --- | --- | --- |
| Caspase-3 | F | TCATAAAAGCACTGGAATGACATC |
|  | R | TTCTGAAATGTTTCCCTGAGGTT |
| Col1A1 | F | AGGGTCTAGACATGTTCAGCTTTGT |
|  | R | GTGATTGGTGGGATGTCTTCGT |
| E-cadherin | F | CATGAGTGTCCCCCGGTATC |
|  | R | TCAGCCGCTTTCAGATTTTCA |
